# Hyaluronic Acid: From Biochemical Characteristics to its Clinical Translation in Assessment of Liver Fibrosis

**DOI:** 10.5812/hepatmon.13787

**Published:** 2013-12-14

**Authors:** Sahar Rostami, Hadi Parsian

**Affiliations:** 1 Student Research Committee, Babol University of Medical Sciences, Babol, IR Iran; 2 Department of Biochemistry, Faculty of Medical Sciences, Tarbiat Modares University, Tehran, IR Iran; 3 Cellular and Molecular Biology Research Center, Babol University of Medical Sciences, Babol, IR Iran; 4 Department of Biochemistry and Biophysics, Babol University of Medical Sciences, Babol, IR Iran

**Keywords:** Hyaluronic Acid, Fibrosis, Liver Cirrhosis

## Abstract

**Context:**

Hyaluronic acid (HA) is a high molecular weight polysaccharide that is distributed in all bodily tissues and fluids. The liver is the most important organ involved in the synthesis and degradation of HA. Research has shown that liver cell injury can affect serum HA levels. In this review, authors aimed to describe the biochemical and physiological roles of this glycosaminoglycan and its changes in various liver diseases.

**Evidence Acquisition:**

Liver fibrosis and in more severe form, cirrhosis are results of an imbalance between fibrogenesis and fibrinolysis. Liver biopsy is the gold standard to assess liver necro inflammatory injuries. This method is invasive and has some major side effects; therefore it is an unfavorable method for both physicians and patients. Now, a wide variety of noninvasive methods have been introduced based on evaluating serum level of different markers. They are safe, readily available, and more favorable. Serum HA levels are used by some researchers to assess stages of liver fibrosis.

**Results:**

There are several scientific studies indicating HA as a biomarker for high score fibrosis and cirrhosis in various liver diseases alone or in algorithm models. It seems from various algorithm models that the use of HA as a major constituent has more diagnostic reliability and accuracy than the use of HA alone.

**Conclusions:**

Use of HA in an algorithm model, is an extra and valuable tool for assessing liver necro inflammatory injuries- in parallel with liver biopsy- but more comprehensive studies are needed to approve the use of HA as an appropriate clinical tool.

## 1. Context

Because of the limitations of liver biopsy in liver fibrosis estimation, various noninvasive tests are presented to assess liver fibrosis stage. In addition to various imaging tests, there are various serum parameters proposed for this purpose. One of the oldest serum parameters is hyaluronic acid (HA). In this paper, we briefly reviewed the biochemical and physiological roles of HA in the human body. In addition, we presented some explanations regarding its changes in various liver diseases. Finally we collected the evidences related to HA clinical application to estimate liver fibrosis. 

## 2. Evidence Acquisition

### 2.1. Hepatic Fibrosis 

Hepatic fibrosis is a wound-healing process in response to an acute or chronic liver injury to parenchymal cells. Cirrhosis is considered the end stage of chronic liver disease, and is able to influence blood flow and hepatic function (1). The most well-known causes of liver disease are various viruses such as hepatitis A, B, and C, hepatic immune disease, alcoholic or nonalcoholic steatohepatitis, inherited metabolic disorders such as Wilson’s disease and haemochromatosis, neonatal liver disease, schistosomiasis, and drug toxicity (1, 2). 

Different liver diseases can make different patterns of fibrosis (3). The process of change from fibrosis to cirrhosis and emergence of clinical symptoms may usually occur after a decade. Progression of fibrosis to cirrhosis is rapid in some conditions. This phenomenon is common in neonatal liver disease, hepatitis C reinfection after liver transplantation, HIV, and hepatitis C virus (HCV) coinfection (1, 3).

One-third of nonparenchymal cells in the liver are hepatic stellate cells (HSC) which exist in the subendothelial space of Disse (1, 3). In chronic liver damage, HSCs undergo a series of changes known as "activation", in which HSCs are transdifferentiated to myofibroblasts, those able to make proliferation, fibrogenesis, and contractility ability (1). HSC activation is the result of an imbalance between extracellular matrix (ECM) synthesis and degradation and the effect of other cellular factors such as cytokines. We can divide this phenomenon into two phases: initiation and perpetuation. In the initiation or pro-inflammatory phase, changes in gene expression would occur and cells would become susceptible to cytokines and stimulus. In the perpetuation phase, fibrogenesis would occur. Other cells such as hepatocyte and sinusoidal endothelial cells are also present in Disse space (1, 3). Liver sinusoidal endothelial cells are functional and important cells in hepatic fibrosis. These cells are able to synthesize fibronectin in very early liver injury and activate HSC. These cells are also able to produce type IV collagen, proteoglycan and some factors that activate the transforming growth factor (TGF-β). TGF- β is a fibrogenic factor produced by many sources, but the bulk of it is expressed autocrine (1). In a physiological condition, nonfibrillar collagens (types IV and VI), proteoglycans such as heparan sulfate, and glycoproteins are the main constituents of ECM. In a pathological condition, due to the effects of fibrogenic factors, fibrillar collagens such as collagen types I and III, noncollagenous glycoproteins such as laminin, fibronectin, undulin, entactin, vitronectin, tenascin, osteonectin and elastin, proteoglycans such as heparin, dermatan and chondroitin sulfates, and various glycosaminoglycan such as hyaluronic acid are replaced and fibrosis would occur (4, 5).

Estimation of the hepatic fibrosis stage is not only helpful in diagnosing the severity of a liver disease, but also in following the patients during the treatment (2, 5). A brief explanation of the most important techniques currently in use for liver fibrosis estimation, are presented here. These techniques are divided into two major classes: invasive and noninvasive ones.

### 2.2. Liver Biopsy

Liver biopsy has been the gold standard for describing liver histology and deciding on treatment options (1, 2, 6). This technique is a valuable method but has some major side effects; the most common are bleeding in the liver and pain around the biopsy area. Others include sampling from a tiny fraction, lack of manpower to undertake several biopsies, mortality rates, and subjective estimation of fibrosis among pathologists (2, 5, 7). Pathologists evaluate biopsy samples by a variety of systems. Ishak, METAVIR and Knodell scoring systems are the most commonly used for grading and staging liver fibrosis and inflammation (2, 6, 8). Today, a wide variety of noninvasive methods are also available. They are safe, easy and currently being validated for diagnosis of fibrosis, cirrhosis and other liver diseases. These include various imaging tests, biochemical and hematological markers and indices. 

### 2.3. Imaging Test

Radiographic tests include CT scan, magnetic resonance imaging (MRI), ultrasound, positron emission tomography, and transient elastography (TE). These tests can provide evidence to evaluate liver fibrosis and portal hypertension. Transient elastography (Fibroscan) measures liver stiffness and predicts the stage of fibrosis. This method is capable of measuring liver stiffness in a volume that is 100 times greater than biopsy samples, and has a positive predictive value of approximately 90% for direct measurement of advanced fibrosis. TE, however can be affected by serum alanine aminotransferase (ALT) levels, so a supplementary noninvasive test independent of serum ALT and/or aspartate aminotransferase (AST) can be helpful in diagnosis (1, 2, 7, 9).

### 2.4. Serum Markers

As previously mentioned, physicians and patients prefer to avoid a liver biopsy and evaluate liver fibrosis noninvasively (6). Studies on evaluating liver disease by serum markers date back to 1970. N terminal propeptide of type III collagen (PIIINP) was the first serum marker suggested for liver fibrosis grading (10). The list of serum markers is long, the most common include; AST, ALT, bilirubin, alkaline phosphatase, albumin, prothrombin time, gamma glutamyltransferase, haptoglobin, apolipoprotein A1, α-2-macroglobin; collagen markers of type I, type II, type III, procollagen I carboxyl terminal peptide (PICP), procollagen IV C peptide, procollagen IV N peptide (7-S collagen), collagen IV, collagenases (metalloproteinase) and their inhibitors (tissue inhibitors of metalloproteinase), glycoproteins such as human cartilage glycoprotein (YKL-40), fibronectin, laminin, osteonectin, tenascin, glycosaminoglycans such as perlecan, hyaluronic acid, decorin, aggrecan, lumican, and fibromodulin (2, 9, 11).

Researchers have also combined the results of panels of individual markers and proposed various algorithms. Some of the most common include; AST to ALT ratio, AST to platelet ratio index (APRI), age-platelet index, PGA index, Forns, Bonacini, PATEL, Leroy, FibroSpect, European Liver Fibrosis score, Fibrometer, Hepascore, SHASTA Index, FIB-4, SteatoTest, NAFLD Fibrosis Score, cirrhosis discriminate score, BARD score, Hui model, FibroMeter NAFLD, Fibrosis Probability Index, Lok Index, and Fibro Q (9, 11, 12).

This review describes the biochemical and physiological role of HA, explains its changes in various liver diseases, and discusses the probable clinical application of HA measurement to assess liver fibrosis.

### 2.5. A Brief History and Structure of HA

Karl Meyer and his colleague John Palmer were the first scientists to discover hyaluronic acid at Columbia University in 1934. In their research, they isolated the chemical substance from the vitreous of cows eyes, and found uronic acid as one of its constituent molecules. They derived its name from hyalos and uronic acid (13). Since 1950, HA has been used as a medical application for eye surgery, which was the first clinical application of this glycosaminoglycan. Later the clinical application of this molecule was described in various fields of medicine, for example in orthopaedy, dermatology, cardiovascular disease and cancer. Since 1985 HA has been considered a sensitive factor in the assessment of liver disease stages (13, 14). 

**Figure 1.**
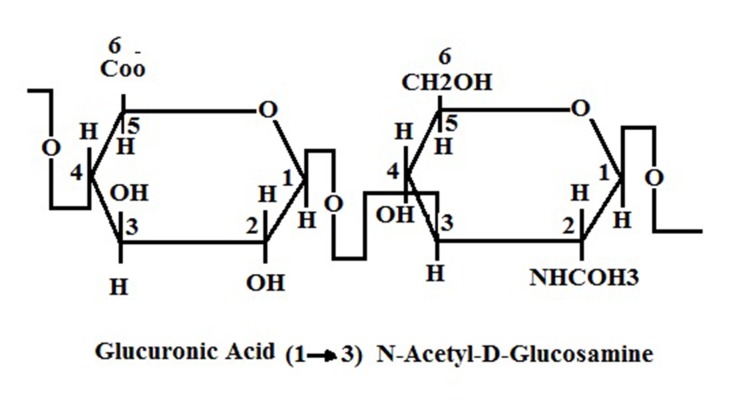
Structure of HA. As it Is Clear, There Is a Glycosidic Bond Between Glucuronic Acid and N-Acetylglucosamine Molecules ( 20 ).

HA is a high molecular weight, nonsulfated, linear chain glycosaminoglycan also known as mucopolysaccharide. This molecule is present in extracellular, pericellular and intracellular spaces. It is composed of a repetitive sequence of hexuronic and amino sugar with acetyl groups [(1→3)-β-D-GlcNAc-(1→4)-β-D-GlcA] (Figure 1) (7, 8, 15). The number of disaccharide in each molecule is 2000-25000; thus its molecular weight can be 105-107 Da (16). The HA molecule is an anionic chain glycosaminoglycan (17). This glycosaminoglycan is a major component of connective tissue, such as the umbilical cord, synovial fluid, skin, and the vitreous body (18). HA exists in different parts of the body in various shapes, sizes and concentrations: freely in the lymphatic system and blood stream, in ECM and also bound to specific receptors on cell surfaces (19). Nearly, a half of HA is present in the skin structure, and a quarter in the skeleton, joints and ligaments. The rest is distributed between other organs, e.g. muscle, lung, brain, liver, and kidney (18).

### 2.6. Biosynthesis and Degradation of HA

HA has some special characteristics that differentiate it from other molecules. These differences include the presence of sulfate group, short sizes of molecular chains, and the biosynthesis pathway. Most glycosaminoglycans are made in endoplasmic reticulum and golgi bodies (18), but HA is synthesized by special enzymes that are located on the inner surfaces of plasma membranes in some tissue, such as the synovial lining cells or hepatic stellate cells. These enzymes are called hyaluronic acid synthases and have three different isoforms in vertebrate, abbreviated as 1, 2, 3 (16). These enzymes are able to synthesize this molecule by adding the activated form of the substrates, i.e. UDP–glucuronic acid and UDP-N- acetylglucosamine, to the growing chain. The resulting molecules are passed through the plasma membrane and ultimately secreted into the extracellular space (13, 21). Mesenchymal cells are the most important cells responsible for the synthesis and secretion of HA into the blood stream. The liver is the major tissue for both removal and synthesis of the circulating form of this macromolecule. In the liver, HA is synthesized by Ito cells and finally degraded by sinusoidal endothelial cells (14, 15). The amount of HA in the thoracic lymph duct is higher than circulating blood stream, because the turnover status of this molecule is very fast and its clearance and degradation would occur rapidly (22, 23). HA degradation can occur locally, for example in skin and joints. 20-30% of HA turnover occurs in situ and the lymphatic system drains the rest. We should consider that HA half-life varies between organs as well as different species. The HA half-life is 2-5 minutes in blood, < 1 day in skin, and 2-3 weeks in cartilages (22, 23).

**Figure 2.**
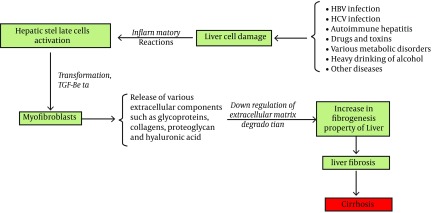
A simple Model for Explanation of Various ECM Components Elevation Including HA in Liver Fibrosis, This Model is Resulted From the Reference (26 )

Among the various organs involved in the degradation of HA, the liver is one of the most important. As previously mentioned, sinusoidal endothelial cells are a major class of cells involved in various metabolism reactions in the liver. These cells also contribute to the rapid elimination of HA from the blood stream (18). During a liver cell injury, the serum HA level rises. Transformation of stellate cells to myofibroblasts, release of various ECM components such as elastin, collagens, glycoproteins, and proteoglycans and HA are later events (Figure 2) (23, 24). Portal vein pressure, level of sinusoids capillarization, sinusoidal flow and intrahepatic shunting are other factors affecting serum HA levels (14). In addition to the liver, HA elimination occurs in the kidney and the spleen (16). Hyaluronidase (hyaluronoglucosaminidase), β-D-glucuronidase, and β-N-acetyl-hexosaminidase are the main enzymes involved in HA catabolism (13, 25). Hyaluronidase hydrolyzes the β 1-4 glycoside bond between N-acetyl-D-glucosamine and D-glucuronic acid, and makes fragments of different sizes (21).

### 2.7. Clinical Applications of HA

According to recently published articles, there are some controversial reports related to the clinical applicability of serum HA in various liver diseases, including hepatic immune disease, alcoholic or nonalcoholic steatohepatitis, hepatitis B, C, and others (26). We have classified the results according to the most common types of fibrotic liver disease.

### 2.8. HA and Hepatitis C

According to the literature, some investigators believe that we are able to differentiate cirrhotic and noncirrhotic conditions (in patients with chronic hepatitis) by serum levels of HA, but some studies did not concur (9, 27, 28). In a study of hepatitis C patients, researchers reported that patients in later stages of liver fibrosis had higher serum HA levels (8). Guechot et al. reported that HA is an important marker for predicting cirrhosis in HCV patients. They reported an HA cutoff value of 110 µg/L with 79% sensitivity and 89% specificity to diagnose cirrhosis vs. fibrosis (27). Other investigators reported other cutoff values for this molecule. McHutchison et al. observed that a cutoff value of < 60 µg/L is the best way to exclude cirrhosis and/or advanced fibrosis. They indicated that one-third of patients with liver cirrhosis have been predicted by the ≥ 60 µg of HA level. In their final conclusion they reported that HA cannot replace the liver biopsy, and histological findings are more reliable (6). Halfon et al. proposed two cutoff values to identify the absence or presence of each stage of HCV (see Table  for more details) (8). In another study by Arain et al. on hepatitis C patients, it was reported that HA is not a reliable marker for selecting treatment decisions (28).

**Table.** Clinical Application of Serum HA Concentrations in Some Recently Reported Papers

Etiology	Number of cases	Stage	Cut off	AUC ^a^	Se, % ^a^	Sp, % ^a^	NPV, % ^a^	PPV, % ^a^	Conclusion	Reference
**HIV/HCV**	201	F ≥ 2	430 ng/mL	-	94.9	15.5	68.4	61	HA is better than other simple noninvasive indices to diagnosis cirrhosis.	(5)
			1250 ng/mL	-	57.3	72.6	55.0	74.4		
			1800 ng/mL	-	30.8	95.2	49.7	90.0		
		F ≥ 3	1800 ng/mL	-	95.3	35.8	94.2	40.9		
			687 ng/mL	-	73.4	68.6	84.7	52.2		
			2290 ng/mL	-	28.1	94.9	73.9	72.0		
		Cirrhosis	1182 ng/mL	-	95.7	56.2	99	22		
			1320 ng/mL	-	91.3	64.6	98.3	25.0		
			2400 ng/mL	-	47.8	94.9	93.4	55		
**HCV**	405 (151 training set, 254 validation set)	F0F1	< 16 µg/L		91	36	82	55	Absence of fibrosis	(8)
		F2	> 121 µg/L		14	99	57	94	Presence of fibrosis	
		F0F1F2	≤ 25 µg/L		78	53	89	34	Absence of sever fibrosis	
		F3	> 160 µg/L		22	100	81	100	Presence of sever fibrosis	
		F0F1F2F3	≤ 50 µg/L		100	79	100	20	Absence of cirrhosis	
		F4	≥ 237 µg/L		31	99	96	57	Presence of cirrhosis(Note: correspondences AUC were: F3F4 = 0.77)	
**NAFLD**	112	Liver fibrosis	≥ 43 ng/mL	0.797	65.7	90.5	61.3	92	HA is useful to discriminate NASH and fatty liver	(35)
		Severe fibrosis	≥ 50 ng/mL	0.797	68.8	82.8	77.9	75		
**NAFLD**	52		19.1 ng/mL	0.672	84	55	86	52	HA is a good marker to predict liver Fibrosis.	(38)
**Chronic hepatitis B (CHB), Chronic hepatitis D (CHD) **	109		64 ng/mL	0.771	70.7	77.6			HA can provide important information to predict liver fibrosis.	(10)
**HCV after transplantation**	46		≥ 90 µg/L	0.89	80	80	89	67	HA could accurately predict the subjects at risk for rapid fibrosis progression after liver transplantation.	(40)
**HBV**	93		126.4 ng/mL	0.98	90.9	98.1	98.1	90.9	HA is the best predictor of extensive fibrosis.	(29)
**Heredity haemochromatosis (HH)**	56		46.5 ng/mL	1	100	100			Measurement of HA in HH patients with serum ferritin >1000 µg/L is an important indicator of the presence of cirrhosis.	(42)
**Viral hepatitis/ HIV**	1252		100 ng/mL	0.83	69.03	87	97.5	27.6	HA was a strong predictor for later development of hepatic encephalopathy or liver related death in HIV co infected HBV or/and HCV.	(41)
**All**	93 children		˃ 50 ng/mL	0.69	65	68	86	40	HA is a valid noninvasive predictor of histological fibrosis in children with liver disease.	(52)
**CHB**	137	moderate to severe fibrosis	≥ 300 ng/mL together with APRI ≥ 1.5		45.3	98.9	91.3	93.7	The APRI ≥ 1.5 in combination with HA cut off ≥ 300 ng/mL is useful to detect moderate to severe form of fibrosis.	(31)
**NAFLD**	79		46 µg/L	0.89	85	80	96	51	Measurement of HA is useful to identify NAFLD patients with severe fibrosis	(36)
**CHC**	22	Significant fibrosis	103.1 ng/mL	0.78	66.7	90	62.2	89	HA showed moderate accuracy to diagnose significant fibrosis, while it seems to be a useful tool to detect advanced fibrosis	(30)
		Advanced fibrosis	109.7 ng/mL	0.92	100	82.3	100	62.5		
**CHB**	93	Mild fibrosis	< 113 ng/mL		92	95	89	94	HA is well correlated with the stage of liver fibrosis and can reflect the severity of liver fibrosis	(48)
		Severe fibrosis	> 181 ng/mL		100	95	100	78		
**CHC**	98	Overall significant disease	20 ng/mL		74	52	71	56	HA might not be regarded as a reliable marker for making treatment decision due to its low NPV. (The authors reported an AUC of 0.716 for overall significant disease).	(28)
			40 ng/mL		41	90	65	77		
			60 ng/mL		28	96	62	85		
			120 ng/mL		22	100	61	100		
**Hepatitis C and end stage of renal disease undergoing haemodialysis**	23		984.8 ng/mL	0.808	83	70			HA is an accurate noninvasive marker to predict significant fibrosis.	(49)
	29		222.3 ng/mL	0.745	74.5	70				
**HBV **	98	Cirrhosis Diagnosis of chronic hepatitis	> or = 154 ng/mL	1	90	100	90	100	HA is a strong tool to predict liver fibrosis.	(50)
**HCV**			> 64.7 ng/mL	0.75	36	100				
**CHC**	49		≥ 65 µg/L		37.5	85.4	87.5	33.3	There was an association between liver function test, HA and liver fibrosis.	(51)
**NAFLD**	100	Any degree of liver fibrosis (F1, F2 versus F0)	1200 ng/mL	0.88			53	90	HA is a predictor of fibrosis in NAFLD children	(33)
		Significant liver fibrosis (F21 versus F0 and F1)	2100 ng/mL	0.95			91	40		
**CHB**	35		52 ng/mL	0.962	91.4	80	84.2	88.8	Serum HA level could be used as an additional clinical tool to evaluate liver fibrosis.	(32)

^a^ Abbreviations: AUC, area under the curves; se,sensitivity; sp, specificity; NPV, negative predictive value; PPV, positive predictive value.

Generally, in comparison with other serum markers such as PIIINP and various types of collagen, HA has a more significant efficacy to predict cirrhosis (21). The ability of HA and PIIINP to differentiate patients with severe liver fibrosis from those with mild liver fibrosis was investigated in another study, and the researchers concluded that HA has more sensitivity and specificity than PIIINP (4). In another study on discriminating those with and without cirrhosis, HA was the most efficient index among HA, N-acetyl-β-D-glucosaminidase, glucuronic acid, glucosamine and AST/ALT ratio (12). The applicability of serum HA levels to estimate liver necroinflammatory injuries, has also been approved in other studies (10, 29).

It seems that the assessment of liver fibrosis by multiple serum markers provides more accurate results than a liver biopsy. In 2002, a systemic review compared the use of single marker, multiple marker and liver biopsy results, and demonstrated that multiple marker results have the greatest diagnostic accuracy (2). Valva et al. showed that the combination of serum levels of HA, PIIINP and TGFβ is more reliable to evaluate the degree of liver fibrosis in comparison with each marker alone (30).

### 2.9. HA and Hepatitis B

The reported studies regarding the usefulness of serum HA measurement in patients with hepatitis B are less controversial, and it seems that many researchers have found a positive correlation between serum HA levels and the stage of liver fibrosis. It seems that serum HA is a more sensitive marker than other serum markers to evaluate liver necro-inflammatory injuries (29). Montazeri et al. showed that serum HA is a more useful marker to estimate the severity of fibrosis stages and inflammation grades than other variables. They observed that the cutoff point of 126.4 ng HA/mL could be used to discriminate advanced fibrosis from mild fibrosis. These results were also confirmed by others (29, 31). There are some reports that used a combination marker model instead of the single marker method. Zhang et al. reported that an APRI score greater than 1.5 when combined with serum HA level (cutoff ≥ 300ng/mL) is a sensitive marker to detect moderate to severe forms of fibrosis (31). Parsian et al. suggested that the level of serum HA and laminin (LN) increased drastically in CHB patients compared to healthy individuals, so it can give valuable information about liver fibrosis progression and also treatment proceedings. They reported a significant association between fibrosis stages, but not inflammation grades, and serum HA and LN levels (32). Seven et al. measured nine serum fibrosis markers including tissue inhibitor of metalloproteinase 1 (TIMP-1), tenascin-C, PIIINP, laminin, matrix metalloproteinase-2 and 9, collagen type IV, collagen type VI and hyaluronan to predict advanced liver disease in patients with chronic hepatitis B. They observed that levels of TIMP1 and HA and finally their combination are powerful markers amongst other measured serum fibrosis markers to be used instead of a liver biopsy (10). 

### 2.10. HA and Alcoholic, Nonalcoholic Fatty Liver Disease (NAFLD) and Nonalcoholic Steatohepatitis (NASH)

NAFLD and NASH are two other important liver diseases. Nobili et al. evaluated the association of some serum markers and liver fibrosis on 100 NAFLD children. In their study serum levels of ALT, AST, gamma-glutamyltransferase, glucose, insulin, and HA were measured. In addition researchers calculated the homeostasis model index of insulin resistance (HOMA-R) as the following formula; fasting insulin [µU/mL] × fasting glucose [mmol/L]/22.5. They concluded that HA is a strong predictor of fibrosis amongst the mentioned serum markers (33). Pares et al. evaluated the status of serum HA and aminoterminal propeptide of collagen III (PIIIP) levels in patients with alcoholic liver disease. They reported that the levels of both HA and PIIIP increased with the severity of liver fibrosis, but only HA levels were related with liver inflammation. In addition, a linear and direct correlation was observed between HA and PIIIP. They concluded that the measurement of serum HA level could be considered a noninvasive test to evaluate the severity of fibrosis (37). Sakugava et al. reported that the domain of serum HA and the type VI collagen 7 had the best correlation with liver fibrosis in patients with NAFLD in comparison with other markers (35). Suzuki et al. suggested that the measurement of serum HA in NAFLD patients is a useful tool to assess liver fibrosis (36). Here also, some studies suggested that a combination of markers is more useful than a single marker to assess liver fibrosis in alcoholic or nonalcoholic steatohepatitis (37). For example, it is proposed that cytokeratin 18 in combination with serum HA levels could be used to identify the progression of liver disease (38). In another study by Nobili et al. it was reported that the combination of HA, PIIINP and TIMP1 (known as Enhanced Liver Fibrosis (ELF)) is a useful model to estimate liver fibrosis stages in NAFLD patients (11).

### 2.11. HA and Other Diseases

There are various diseases in which the serum levels of extracellular matrix components, including HA, would be changed. Investigators have reported that serum HA levels can be used as an extra tool to discriminate patients with the mild form of schistosomiasis and those with the severe form (14). There are some reports indicating that serum HA is useful to diagnose liver damage after hepatectomy and transplantation. Nanashima et al. observed that serum HA levels increased after a liver resection (39). Pungpapong et al. reported that HA and YKL-40 are the best markers to diagnose patients with chronic hepatitis C and rapid fibrosis progression after liver transplantation, and claimed that this method is better than the histological biopsy (40). Peters et al. in their study on patients with viral hepatitis and HIV coinfection demonstrated that HA is a strong marker to predict liver-related death in HIV/HBV or HCV patients (41). There are some reports concerning the usefulness of serum HA measurements in metabolic diseases. In 2009, Crawford et al. showed that HA measurement is a useful indicator to diagnose cirrhosis in patients with heredity haemochromatosis and serum ferritin > 1000 µg/L (42). It has been shown that after injection of dimethylnitrosamine and induction of liver toxicity, HA levels increased drastically (19, 43). In acetaminophen-induced acute liver damage, interference between the endothelial cell HA receptor and the toxic product could increase serum HA levels (44).

Tangkijvanich et al. measured the serum levels of HA in patients with hepatocellular carcinoma (HCC), and observed that the level of HA in HCC patients was significantly higher than those of the normal group (426.3 ± 687.33 vs. 117.86 ± 311.11 ng/mL, respectively) (24). In a study by Sadik et al. on HCC in patients with and without cirrhosis, they determined serum levels of adiponectin and HA and compared these values to normal subjects. They observed that levels of serum adiponectin and HA were higher in patients compared to normal subjects (45).

## 3. Results

We have summarized the results of above mentioned studies in a (Table) with more statistical details. In this Table the etiology of disease, liver fibrosis stage, best reported cutoff value, related parameters regarding receiver operating characteristic curve (ROC), area under the curves (AUC), sensitivity, specificity, positive and negative predictive values are presented. 

## 4. Conclusions

There are several scientific studies indicating HA as a biomarker for high score fibrosis and cirrhosis in various liver diseases. It seems from various algorithm models that the use of HA as a major constituent has more diagnostic reliability and accuracy than the use of HA alone. Some researchers have named HA a hepatic fibrosis marker, but it seems that fibrosis index may be more appropriate. HA is not a specific marker solely to diagnose liver disease, since its elevation is known in other situations, such as rheumatoid arthritis, pulmonary fibrosis, connective tissue disorders, psoriasis, scleroderma and gastrointestinal, prostate, bladder, and other cancers (24, 41, 46). In addition diurnal variations, age, exercise and food ingestion are known as effective factors on serum HA levels (15). Thus any increase in blood HA level is not a definitive indicator of the presence of liver necroinflammatory injuries or other diseases. 

In conclusion, it seems that use of HA, alone or in an algorithm model, is an extra and valuable tool for assessing liver necroinflammatory injuries, in parallel with liver biopsy. Surely, more comprehensive studies are needed to approve the use of HA and other related indices as an appropriate and precise clinical tool for liver necroinflammatory injuries assessment (34). 
